# Vision based supervised restricted Boltzmann machine helps to actuate novel shape memory alloy accurately

**DOI:** 10.1038/s41598-021-95939-y

**Published:** 2021-08-12

**Authors:** Ritaban Dutta, Cherry Chen, David Renshaw, Daniel Liang

**Affiliations:** 1 CSIRO DATA61, Hobart, Australia; 2 CSIRO Manufacturing, Clayton, Australia

**Keywords:** Materials for devices, Soft materials, Techniques and instrumentation, Theory and computation

## Abstract

Extraordinary shape recovery capabilities of shape memory alloys (SMAs) have made them a crucial building block for the development of next-generation soft robotic systems and associated cognitive robotic controllers. In this study we desired to determine whether combining video data analysis techniques with machine learning techniques could develop a computer vision based predictive system to accurately predict force generated by the movement of a SMA body that is capable of a multi-point actuation performance. We identified that rapid video capture of the bending movements of a SMA body while undergoing external electrical excitements and adapting that characterisation using computer vision approach into a machine learning model, can accurately predict the amount of actuation force generated by the body. This is a fundamental area for achieving a superior control of the actuation of SMA bodies. We demonstrate that a supervised machine learning framework trained with Restricted Boltzmann Machine (RBM) inspired features extracted from 45,000 digital thermal infrared video frames captured during excitement of various SMA shapes, is capable to estimate and predict force and stress with 93% global accuracy with very low false negatives and high level of predictive generalisation.

## Introduction

Newly discovered SMAs are increasingly being used for application solutions in automotive^1^, aerospace^2^, construction and other commercial fields^3,4^ for their extraordinary ability to fully recover to their original shape from an actuated shape. The unique capability to memorize the original structural shape and recovery back to that shape from an excited state, has made this type of materials a novel candidate for the future generation soft robotics and for the applications with a requirement of lightweight or miniaturised actuation^5–8^. Recovery characteristics of this SMA provide a superior level of flexibility in movement and higher degree of freedom than moving a conventional mechanical structure, especially in applications where the volume and weight of actuators are restricted.

Thermally activated SMA based actuators are the potential candidates to replace the traditional actuation systems including for applications in space stations, satellites, or planet robots^9^. Compared with the traditional space actuating sub-systems, the SMA actuators have a high power-to-weight ratio, a simple actuation mechanism, no dust particles created or no leakage of fluids, thus reducing the complexity, sizes and the weight of an actuator^10^. The thermally induced SMAs perform shape memory effect (SME) by undergoing solid-state transformations between low and high temperature phases, which are known as martensite and austenite phases, at characteristic temperatures. The shape change intrinsically occurs only during heating from martensite to austenite phases, not cooling from austenite to martensite, referring to one-way-SME, non-reversible. By applying a series of heat treatments or “training” processes, SMA can have two-way-SME with a limited degree of reverse actuation that are of little usefulness^11,12^, e.g., less than 20–30-degree angles for a reversible bending strip^13^. Therefore, most of the commercial SMAs are supplied only for performing a single-point, mostly one-way, actuation—i.e. one-direction and non-reversible motion at one fixed temperature. Consequently, the lack of an effective, reversible actuation ability has limited the use of the SMA actuators to very few simple applications, such as one-off release in QWKNUT and Low-Force Nut (LFN)^14^, locking or deploying mechanisms for truss mounting^15^ folding structures^16^, and deployable hinges of lightweight flexible solar array^17^.

**Figure 1:**
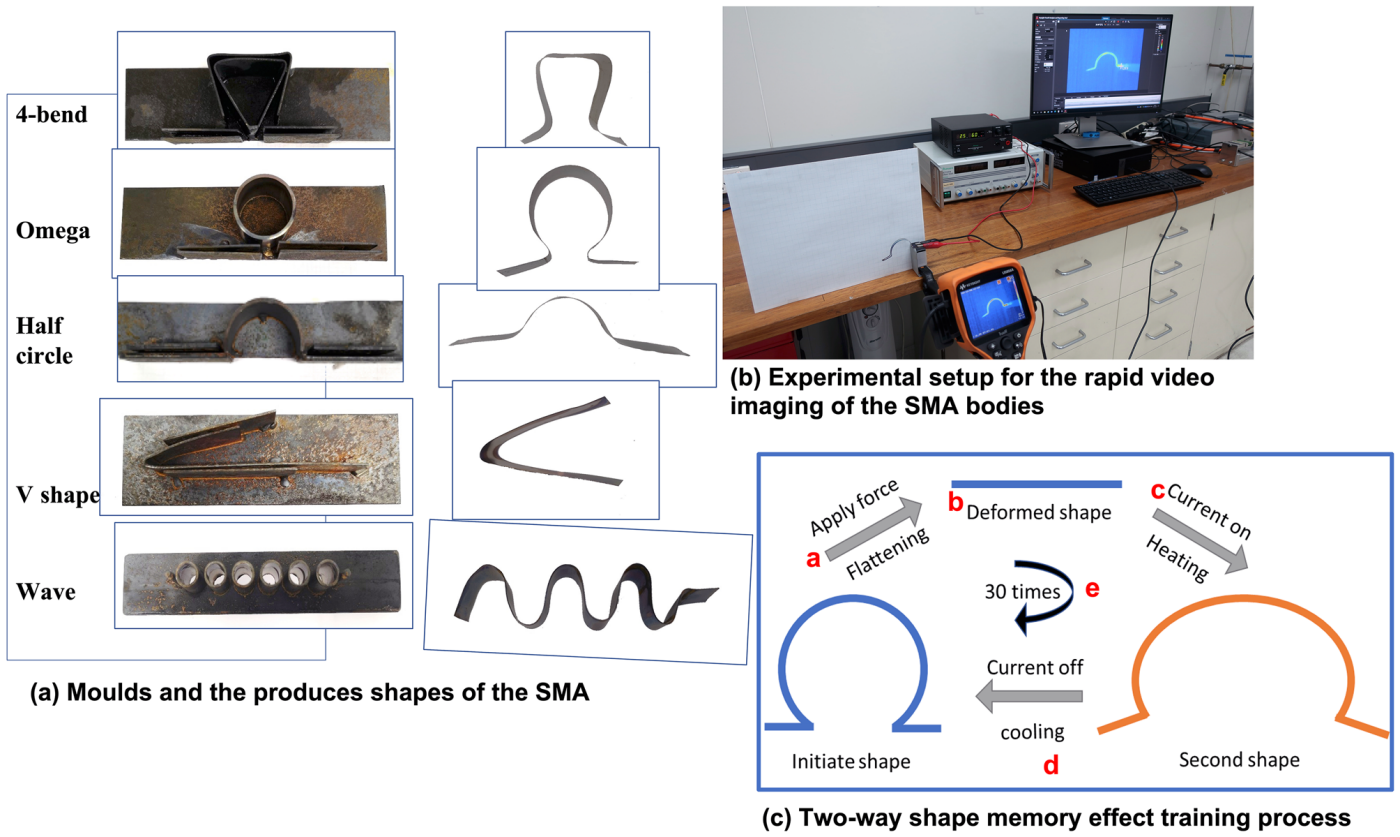
**(a)** Five steel moulds to shape SMA strips into different shapes and the shaped SMA strips in “Omega”, “Half circle”, “V”, “4-bend” and “Wave” shapes; **(b)** experimental setup to generate data for the rapid computer vision approach and machine learning based prediction; **(c)** two-way shape memory effect training process, involving steps marked as a-e to train the body.

The CSIRO scientists have developed a rapid solidification process that can produce NiTi based SMA foils with a larger width that can be utilised for constructing actuators^18^. More importantly, the obtained SMA foils exhibit a larger reversible actuation after thermomechanical cycling. As a demonstrative example of the shape memorisation training of a SMA body, the large-degree reversible actuation of a CSIRO-produced Omega-shaped-NiTi SMA body is illustrated in Fig. 1. Between electrical heating on and off, the Omega-shaped SMA was closing and opening over a 60 degrees angle, showcasing high potential for an industrial adaptation.

This has achieved a new step towards the wider adaptation of SMA materials in various applications. However, despite the current advances in the field of reversible shape memory materials, applications of this family of novel materials remain a unique challenge due to difficulty in accurately controlling and reversibly actuating a SMA body^19^. Like any moving physical body, SMA based structures also generate force while the whole body or a part of the body moves under an external excitement. The actuation amount and speed, thus their control, are influenced by the interaction between the internal actuation force generated at the atomic scale and the resistance to the actuation that is related to bulk materials properties. As the SMA internal interaction of the forces are unique and are difficult to quantify, the characterisation of the forces and their effects on the actuation behaviours through conventional ways either experimentally or analytical modelling are difficult and time consuming^20–22^.

**Figure 2:**
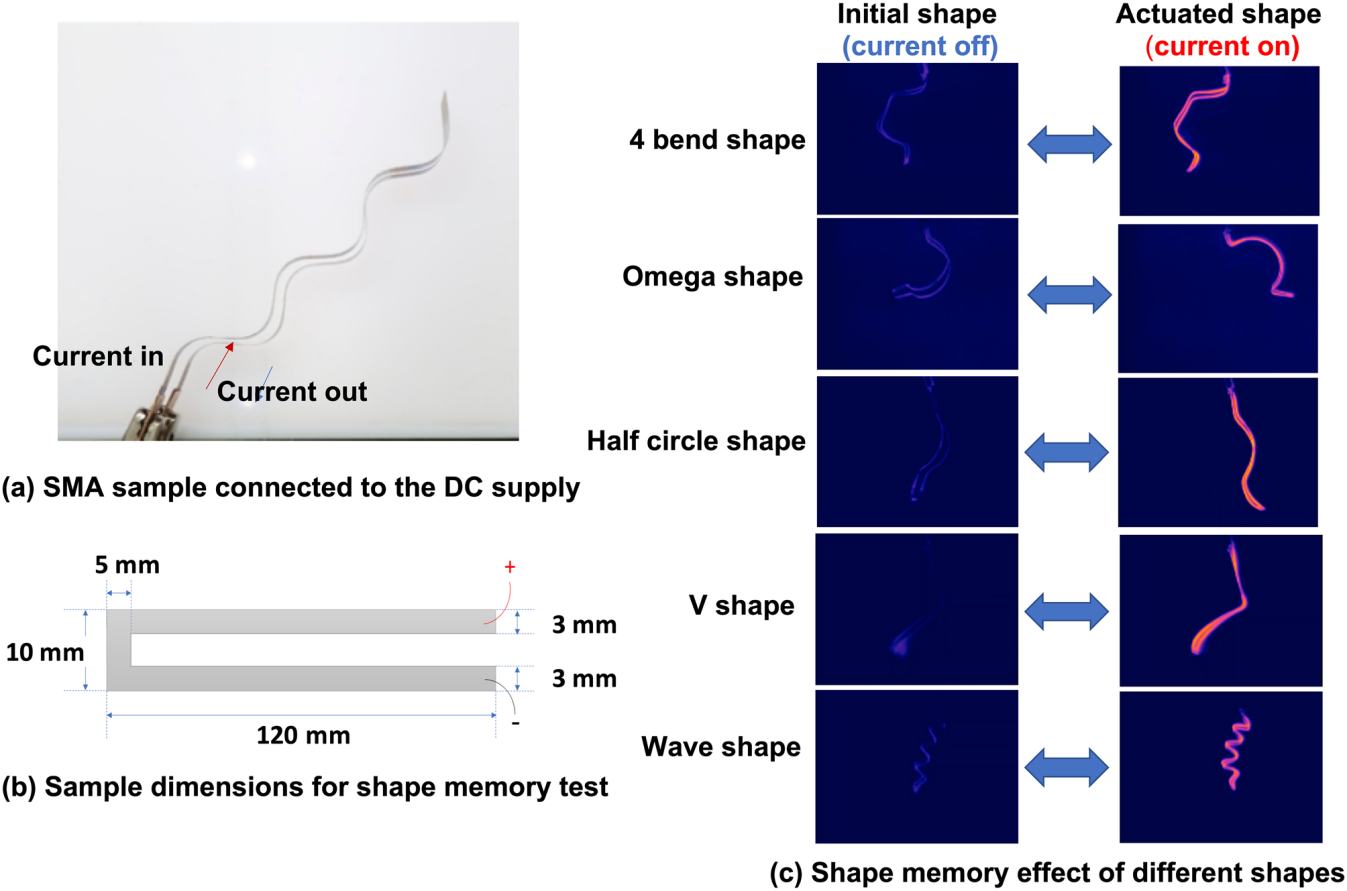
**(a)** Two legs of SMA connected to the DC supply; **(b)** the sample dimension of SMA used in this study; (3) thermal images of the initial and actuated shape of the five different SMA bodies that were used in this study. The changes in shape and position during electrical excitement were the key dynamic information captured through the video imaging. The captured information was correlated with the measured force and stress to develop a predictive machine learning model.

**Figure 3:**
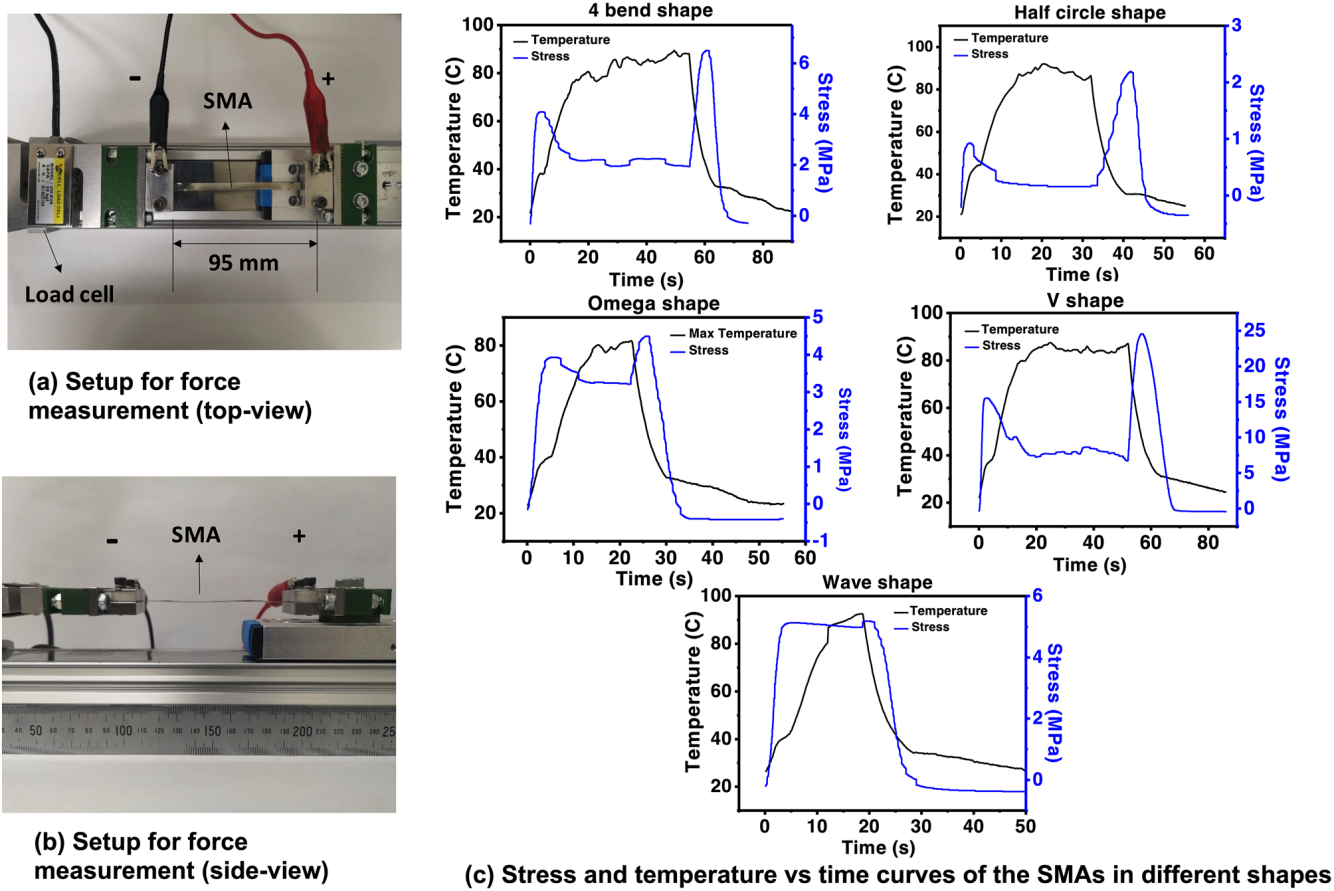
Setup of the force measurement **(a)** top-view and **(b)** side-view. **(c)** Shows the stress and temperature characteristics curves for the five SMA shapes.

The aim of this study was to develop a predictive modelling system in order to actuate the novel reversible SMA materials in a more controllable way. A method to rapidly and accurately measure the amount of the force generated by the SMA movement is the first essential step to control and actuate the SMA body. The second step in undertaking this reported work was to develop an intelligent modelling system for the accurate estimation of the force generated by a group of reversible-actuating SMA bodies of various shapes (as shown in Figs. 2 and 3).

A promising predictive modelling has been proposed and developed in this paper. This combines computer vision and machine learning to characterise novel SMA materials and estimate force generated by a moving SMA body under external excitement. We employed infrared digital camera to capture the video of a moving SMA body, while measuring generated force simultaneously. The measured force was used as benchmarking ground truth data for all future modelling.

The change in the relative position and shape of the SMA body compared to its original position and shape under excitement, was captured in the video frames. This dynamically changing information about shape and position were correlated with the separately measured generated force for using the proposed predictive modelling^23–31^.

**Figure 4:**
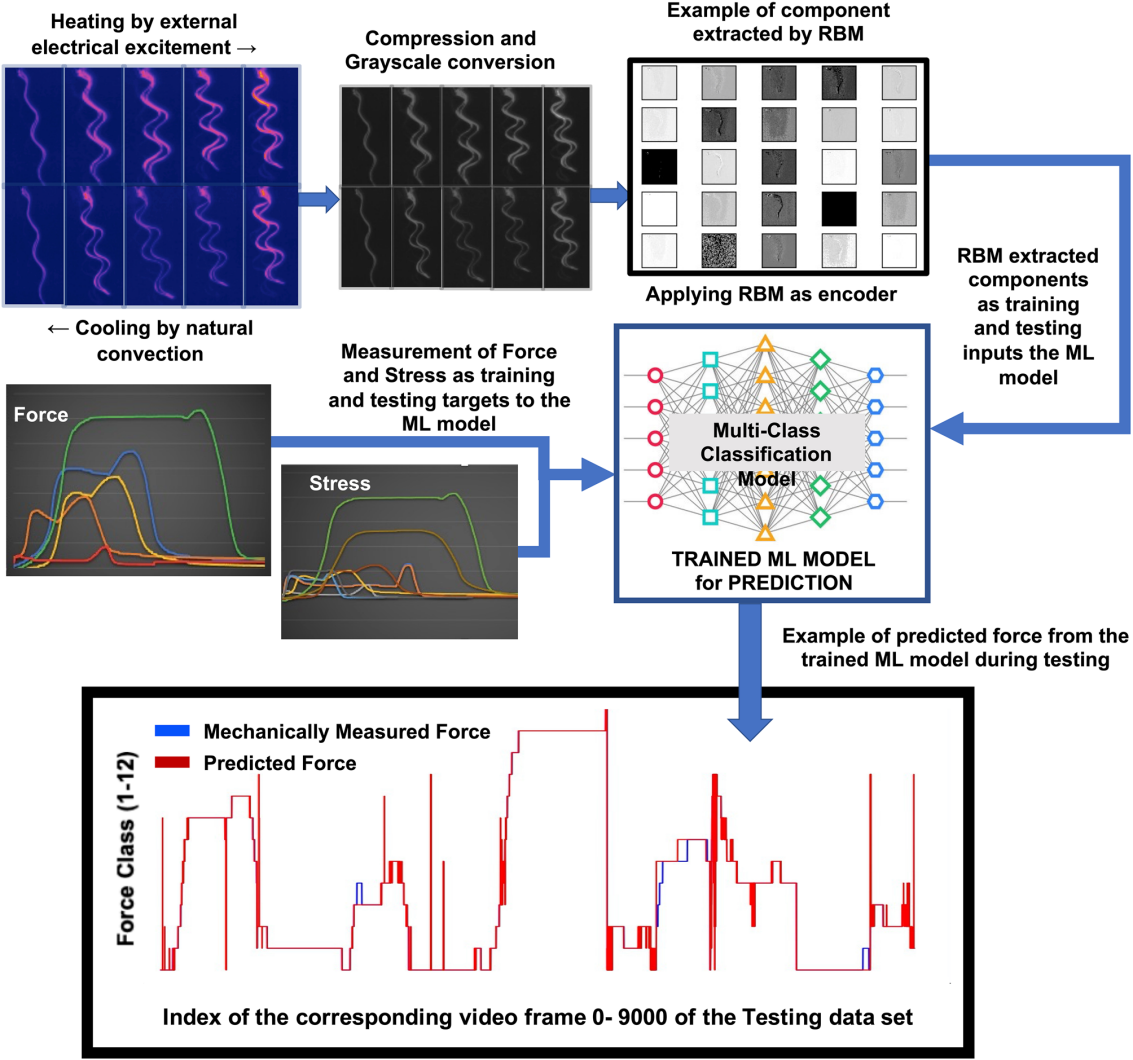
This figure shows the overall computer vision and machine learning based framework that was employed in this study. RBM based feature extraction combined with Random forest classification algorithm was able to improve the force and stress prediction accuracy from 84 to 93%. This framework also demonstrated that a computer vision approach could be used to characterise novel material rapidly. During the testing phase, the experiment mechanically measured ground truth force was compared with the predicted force derived from the proposed computer vision (or optical) method. This comparison was the basic method to derive the overall accuracy of this predictive method.

We chose to use vision based supervised Restricted Boltzmann Machine (RBM) approach combined with a machine learning classifier algorithm to make this estimation. Machine learning algorithm in classification tasks has proven to be highly effective in a wide range of applications while being free of the restrictive assumptions of other predictive systems^32^. RBM has been proven to be a very effective technique to capture unique set of features or components from video data. RBM was used as a feature extraction technique, while extracted features were used to train a machine learning classifier for developing a predictive model capable to estimate and predict the force generated by a moving SMA body. Figure 4 showcases a schematic diagram of the employed machine learning framework involving RBM and a machine learning classifier^33–39^ and prediction validation of such an approach.

## Preparation of the SMA and thermal treatment

In this study, NiTi based shape memory alloy (SMA) foils with a width of 30 mm and a thickness around 80–150 μm, were prepared by planar flow casting i.e. a rapid solidification process. The SMA foils were cut into 10 mm width and 120 mm long strips and were carefully inserted into the slot of a metal mould. The mould with the sample was placed inside a tube furnace, undergoing a standard annealing process at 550 °C for 30 min in an argon atmosphere before quenched in water. In this study, five different shapes of SMA strips were produced. The moulds along with the shaped SMA strips are shown in Fig. 1a. As indicated by the schematic in Fig. 2b, a centre slot 4 mm wide, 115 mm long has been cut out of the SMA strip after moulding. This is to form the electrical current path for actuating the SMA.

### Chemical analysis

The SMA foils were characterized by inductively coupled plasma optical emission spectroscopy Varian 730-ES ICP-OES (ICP-OES). Around 0.1 g of the sample was dissolved in a solution of HNO_3_, H_2_SO_4_, HF and water before the ICP-OES analysis. After calibrated by certified multi-element solutions, the analysis results showed that the SMA consisted of 49.1% of Ni and 50.9% of Ti.

### Thermal analysis

Differential Scanning Calorimetry (DSC) was selected to analyse transformation temperatures of the SMA samples. Around 5 mg of SMA sample was used for the test under a nitrogen flow (40 ml/min). A heating and cooling schedule consisting of a heating rate from − 20 to 100 °C. Followed by a cooling rate from 100 to − 20 °C. Both heating and cooling rates both were at 10 °C/min. DSC thermogram was obtained using a Mettler Toledo DSC3. The transformation temperatures were extrapolated from the DSC data through the tangential line method: Martensite start temperature (M_s_), Martensite finish temperature (M_f_), Austenite start temperature (A_s_) and Austenite finish temperature (A_f_) are 60.8, 42.33, 72.22 and 89.54, respectively.

### Shape memory alloy training

A certain thermomechanical treatment (so-called training process, shown in Fig. 1c) was carried out on the SMA test samples: (a) deform the samples into flat strips; (b) connect each leg of the strip to a DC power supply; (c) apply a current of 7 A at the voltage of 5 V for 10 s. The temperature of the samples increased due to the Joule heating effect and the samples were recovered to their initial shapes; (d) turn off the current and allow it to cool down to room temperature; (e) repeat (a) to (d) steps for 30 times. After this training, the SMA samples “remembered” two status i.e. flat and shaped when the current was on and off, showing two-way shape memory effect.

### Video data capturing and pre-processing to study SME

An infrared thermal camera fixed on a test rig was used to capture the change in temperature, position and shape of the SMA bodies while under electrical excitation as shown in Fig. 1b. The trained SMA flat sample is connected to a DC supply with a current of 7 A and a voltage of 5 V, one leg is for current in and the other is for current out to ensure a close loop (Fig. 2a,b). When the current was applied, the temperature of the SMA was increased and it started to form the trained shape. When the current is turned off, the SMA sample recovers to the original flat shape. The thermal video files were treated as a combination of static frames of size 1200 by 1200 pixels. The individual frame was compressed to 300 by 300 pixels size and converted to grayscale images (as shown in Fig. 4). The quality of the captured video was kept consistent during all experimentation in that 51,000 video frames of the five different moving SMA bodies were collected. Standardizing the experimental protocol was crucial to capture data of a high quality that are consistent and completely reproducible.

The difference between two consecutive video frames (DF) was used as a differential and representative information for capturing the changes in shape and position demonstrated by the SMA body under excitement. The change in shape and position was apparently the key indicator of the force generated. Each of the DF was pre-processed to extract representative features to be used in the training and testing of the predictive machine learning algorithms. Selection of a DF to be included in the final study, was determined by a significance tolerance factor, defined by the image pixel wise difference between two consecutive frames being greater than 5%. This was to eliminate the repetitive video frames (without any significant changes) from the overall analysis and any potential bias that could be created by this type of repetition. Finally, 45,000 DFs were selected to be included in the analysis.

RBM was used in this study for pre-processing of the video data. We found that RBM based feature representation was better suited as an encoder for this study over a conventional autoencoder, as RBM was faster to process the large volume of video frames with standard available libraries. Parameters are estimated using Stochastic Maximum Likelihood (SML), also known as Persistent Contrastive Divergence (PCD)^31–43^. We utilized RBM as a feature extraction method to reduce the very large dimension of the video frames. We found that 150 extracted components by RBM was optimum and best to explain the variance among the video frames to be classified into one of the force classes and stress classes accurately. Optimum learning rate for the RBM was determined to be at 0.412.

### Force measurement

The tester to measure the actuation force is shown in Fig. 3. It is equipped with a 20 kg load cell. The two sample holders of the tester were connected to a DC power supply (DPD3030, Manson). The applied current was 5 A and the voltage was 2.3 V.

The SMA samples were cut into a strip with width of 5 mm and length of 120 mm for force measurement. After thermally treated, shaped and trained, the SMA test samples of different shapes are flattened and placed onto the tester with both ends of SMA sample screwed onto the tester sample holders. Torque wrench was used to tighten the screws, ensuring even clamping force is applied to both ends of sample. The test strip is then stretched along length ways of the SMA sample (or distance between the two tester arms was 95 mm).

During the tests a current was applied to the samples, the temperature of the samples would increase due to the Joule heating effect. The SMA strips would begin to recover to their original shape when they reached their phase changing temperature. As the ends of the samples were restricted by the sample holders, generated force was applied onto the sample holders, then detected by the load cell. Results recorded are.

Measured time series of the force and force per section area (i.e. stress) were time stamped with the corresponding differential video frames (DFs) and the extracted 150 RBM components from each of the DFs.

### Input and output for the machine learning modelling

It was important to note that for this study we aimed to develop a single predictive system for any shape of the SMA, hence all pre-processed data from the various shapes were combined into a single data set. The aim was to test a generalisation capability to predict force and stress while employing computer vision and machine learning algorithm.

The data set included 10,500 related to the “Omega”, 9000 related to the “Half circle”, 9500 related to the “V”, 7500 related to the “4-bend” and finally 8500 related to the “Wave” shaped body under experimentation. The amount of force was represented by a number between 1 and 12 as the measured values, whereas the amount of stress was represented by a number between 1 and 20 as the measured values. They were directly suitable to be utilised as class labels of the proposed machine learning based multiclass classification problem. Altogether the data set had 45,000 data entry points after removing all repetitive DFs with 5% or less variance between two consecutive video frames.

The final data set had 45,000 selected differential video frames (DFs), each of which represented by extracted 150 RBM components, along with an associated measured amount of force and stress collected during five sets of experiments on SMA bodies with five different shapes (Fig. 1).

The dimension of the whole data set was 45,000 rows × 152 columns, where the first 150 columns were representing 150 RBM components (each row representing each of the DFs) and the last two columns were representing the force and stress values associated with each of the DFs.

The machine learning algorithms were trained with RBM components as inputs with force and stress as training target. In this way, during testing and validation, a trained model was prepared to predict force and stress against a set of unknown inputs. The unknown inputs were the extracted RBM components from a portion of the selected DFs which were not a part of the training of the algorithms.

For the predictive modelling using machine learning algorithms, first 150 columns of RBM components were used as training and testing inputs. The last two columns with values of measured force and stress were used as training and testing learning targets. A schematic diagram of the data pre-processing and machine learning based training and testing paradigm has been described in the Fig. 4.

### Machine learning modelling

We designed the proposed system to predict a force and stress value for an individual video frame based on the dynamic movement of a SMA body irrespective of its shape. Hence the problem space was formulated to identify a unique feature pattern representative of a unique range or values of force or stress, which can be learned into a machine learning model. This type of unique feature pattern representing a specific range or values of force and stress, could be derived from any video frame captured from any of the five different types of SMA bodies. Learning a unique set of features against a unique range or values of force or stress was main aim of this modelling.

We found that a multi-class classification approach was more suited over a multi-output regression approach for the machine learning algorithms to learn and predict, while only inputs for the training and testing were the extracted 150 RBM components. In this approach, each of the selected DFs represented by a set of 150 RBM components, only needed two simple class labels as training targets, one for the associated force values and the other for the associated stress values, for the machine learning modelling.

The physically measured force of the 5 shapes at a specific time of the experiment was marked as the ground truth force associated with that DF to be learned as a target in the machine learning algorithm. Physically measured force values were categorised into 12 force classes (e.g., 0–1, 1–2, 2–3…, 11–12) while each class represented a force range 1 Newton gradient) to formulate a quantisation based multiclass classification problem. Similarly, measured stress values were categorised into 20 force classes (e.g., 0–1, 1–2, 2–3…, 19–20), while each class represented a class of stress of range 1 MPa gradient). Idea behind the quantization of the force and stress time series into multi-class representation was to simplify the quantification of the prediction accuracy in a traditional manner. It was also found that machine learning algorithms can be trained more accurately with coded class labels.

The development of the predictive model was based on a multi-output multi-class classification model. With suitable training the same feature space representing the changes in the video frames, a model was able to predict two class labels simultaneously, one for the force (class number ranging between 1 and 12) and other for the resistance stress (class number ranging between 1 and 20). A classification probability estimation system was incorporated in conjunction with the multi-class classification system. Each of the class prediction related to force was associated with 20 class probabilities whereas each of the class predictions related to stress was associated with 12 class probabilities. The class probability estimation provided a confidence measure of the accuracy of the classification. Highest probability value for a particular class represented a clear classification while similar probability values for more than one class represented a borderline classification scenario with a possibility of a miss-classification.

Because there are many possible types of machine learning classifiers, we tried ten types of classifier systems representing a wide range of algorithms. This was aimed to determine the most appropriate and efficient of these classifiers, and to justify the effectiveness of proposed machine learning framework for this study. As a comparative paradigm, namely, Feed Forward Back Propagation, Support Vector Machine, Multi-Layer Perceptrons, Random Forest, Radial Basis Neural Network, Decision Tree, Naive Bayes, Quadratic Discriminant Analysis, Gradient Boosting, Logistic Regression classifiers were applied to the same data sets to establish the best artificially intelligent feature learning architecture as indicated by force and stress estimation accuracy^40–43^.

Initially the whole data set was randomly split into 80%-20% proportional ten combinations. In each of the new subsets, 80% of the data (combination of inputs and targets) was used in the training–testing of the predictive model, while the rest of the 20% of the data was kept separately for a final validation of the trained model. In each of the ten training–testing phases with the 80% of the data, internally, a randomized tenfold cross-validation (CV) technique was adapted to overcome the overfitting. In this well accepted approach in the machine learning domain, called k-fold CV, the training set is split into k smaller sets. The performance measure reported by k-fold cross-validation is then the average of the values computed in the loop. This approach can be computationally expensive but does not waste too much data. Similarly, in each of the ten corresponding validations, mean accuracy was computed to report as a final accuracy estimation from the whole train-test-validate paradigm (Table 1).

**Table 1:** Multioutput multiclass classification results from the ten different machine learning classifiers.

Multioutput multiclass classifiers to predict categories representing class of generated force and resistance stress	Accuracy result based on RBM extracted features		Accuracy result without the RBM extracted features
	10-fold CV mean test accuracy (%) (± standard deviation)	Mean validation accuracy (%) (± standard deviation)	Mean validation accuracy (%) (± standard deviation)
Feed forward back propagation	66.67 (± 11.56)	62.07 (± 13.56)	52.07 (± 18.76)
Support vector machine	73.33 (±10.07)	78.33 (±15.07)	70.21 (±17.34)
Multi-layer perceptrons	76.66 (±15.6)	72.66 (±11.6)	73.66 (±15.2)
Radial basis neural network	89.11(±8.31)	86.56(± 8.31)	79.21(± 11.91)
Random forest	90.37 (±5.07)	92.88 (± 6.77)	84.18 (± 9.06)
Decision tree	87.77 (± 17.77)	80.17 (± 7.77)	73.17 (± 12.77)
Naive Bayes	70.44 (± 10.09)	71.44 (±14.09)	60.74 (±13.79)
Quadratic discriminant analysis	60.00 (±18.32)	65.00 (± 16.32)	55.00 (±18.30)
Gradient boosting	75.55 (±11.16)	73.15 (±12.16)	62.56 (±15.36)
Logistic regression	77.05 (±12.87)	70.55 (± 14.87)	67.55 (±10.47)

A random forest (RF) classifier was the best performing classifier in the multiclass classification problem while predicting the generated force and the stress by a movement of a SMA body. It was found that a restricted Boltzmann machine (RBM) was able to extract features efficiently to enhance the prediction accuracies of the classifiers. These findings led to a predictive system which can help to control and actuate the SMA structures more accurately.

## Results

We can conclude that there are two main reasons for the superior classification performance of the Random Forest (RF) technique compared to Feed Forward Back Propagation, Support Vector Machine, Multi-Layer Perceptrons, Radial Basis Neural Network, Decision Tree, Naive Bayes, Quadratic Discriminant Analysis, Gradient Boosting, and Logistic Regression^43–47^. RF classifier can adapt themselves to the distribution of abstract feature space extracted from a video stream, making them hugely popular as a robust classifier for various applications. RF was able to classify most of the patterns corresponding to force and stress classes, due to their ability to adjust their scale of generalization to match the morphological variability of the patterns in the video frames. RBM based feature extracted from the video frames helped to increase the linear separability of the multiclass classification problem. In Table 1, we also compared results between two scenarios, with and without using RBM features. In the later scenario whole flattened video frame was used as the input vector for the classifying task. It was evident that all the classifiers performed significantly better with RBM features while RF was outperforming other classifiers with an enhanced performance from 84 to 93% improved accuracy.

The performance of the estimators was quantified by the prediction accuracy ((TP + TN)/(TP + FN + FP + TN) where TP = true positives, TN = true negatives, FP = false positives, FN = false negatives). From the achieved mean accuracy from the tenfold CV process, it was found that RNN was the best to handle this classification problem with 93% global accuracy with very low of false negatives. Results for the independent testing and validation are summarised in the Table 2 (for the generated force) and Table 3 (for the resistance stress).

**Table 2:** The complete classification report for the RF based multiclass classification results including prediction accuracy, precision, recall, F1-score of classification results from the independent validation stage while predicting class labels related to the classes of generated force by the SMA body movements.

Force class labels—equivalent of generated measured force	Precision estimation	Recall estimation	Calculated F1-score	Total number of support samples for the individual class
1	0.93	0.98	0.95	1268
2	0.96	0.90	0.93	960
3	0.92	0.89	0.91	608
4	0.94	0.83	0.88	670
5	0.90	0.89	0.89	540
6	0.81	0.92	0.86	580
7	0.84	0.88	0.86	900
8	0.96	0.95	0.96	560
9	0.99	0.97	0.98	506
10	0.83	0.71	0.77	558
11	1.00	0.85	0.92	890
12	0.99	0.98	0.95	960
Accuracy			0.93	9000
Macro Avg	0.91	0.89	0.90	9000
Weighted Avg	0.93	0.93	0.93	9000

**Table 3:** The complete classification report for the RF based multiclass classification results including prediction accuracy, precision, recall, F1-score of classification results from the independent validation stage while predicting class labels related to the classes of stress.

Stress class labels—equivalent of generated stress	Precision estimation	Recall estimation	Calculated F1-score	Total number of support samples for the individual class
1	0.93	0.98	0.95	789
2	0.96	0.90	0.93	678
3	0.92	0.89	0.91	245
4	0.94	0.83	0.88	376
5	0.90	0.89	0.89	560
6	0.81	0.92	0.86	344
7	0.84	0.88	0.86	691
8	0.96	0.95	0.96	230
9	0.99	0.97	0.98	278
10	0.83	0.91	0.92	501
11	1.00	0.85	0.92	457
12	0.99	0.98	0.95	890
13	0.90	0.89	0.89	751
14	0.81	0.92	0.86	220
15	0.84	0.88	0.86	490
16	0.96	0.95	0.96	600
17	0.99	0.97	0.98	210
18	0.90	0.89	0.89	201
19	1.00	0.85	0.92	230
20	0.99	0.98	0.95	259
Accuracy			0.92	9000
Macro Avg	0.92	0.88	0.91	9000
Weighted Avg	0.91	0.92	0.92	9000

Prediction accuracy, precision (defined by TP/(TP + FP)), recall (TP/(TP + FN)), F1-score (defined by 2 × (precision × recall)/(precision + recall)) and classification probability results are reported in these tables. The precision, recall, and F1 score (also F-score or F-measure) are measures of an experiment’s accuracy, where a score of 1.0 indicates perfect classification and a score of 0.0 indicates all examples were incorrectly classified. Tables 2 and 3 shows the complete classification report for the RF based multiclass classification results. Macro and Weighted average of the class-based prediction accuracy were also reported in the Tables 2 and 3.

## Conclusion

Future generations of SMA materials will be designed with highly bio-inspired motivations. They will aim to be soft bodies composed of soft materials, part of soft actuators and sensors, and will be capable of soft movements and safe interaction with humans. Development and wider adaptation of SMA materials will rely on higher degree of freedom in movement and actuation.

we developed a group of novel SMA materials suitable for large degree reversible actuation mechanism. We demonstrated that a newly developed NiTi based SMA with equilibrium microstructure was able to achieve two-way actuation performance. Validation of this development was tested through consistent reversible actuation of various shapes of the same SMA material.

In this work, we reported accurate estimation of generated force and stress through an intelligent system instead of a conventional time-consuming approach. We combined computer vision techniques and scalable machine learning to develop a model-based architecture for rapid characterization of shape memory materials. The reported intelligent system was proven to be highly accurate in computer vision based rapid estimation of force and stress generated by a movement. Using computer vision and machine learning based modelling techniques to model deformable SMA bodies is a unique paradigm shift in the conventional characterisation of novel materials, which is predominantly actuated and controlled by central processing units and motors.

Through various experimental paradigm, we identified that there is a need to employ an intelligent system to characterise this novel material in order to achieve a greater control and intended actuation. Novel SMA material can only achieve a greater milestone in real life application, if combined with a suitable pathway to flexible actuation. It was established that a data driven framework is needed to rapidly estimate force and stress generated by a moving SMA body.

In future this computer vision and machine learning based manufacturing video data processing system could easily be adapted as a standard web based system to create a digital tool for the rapid SMA material characterisation. This enhanced capability is able to capture and process raw data in-situ, so the generated data can be accessed readily for modelling and simulation. The data acquisition capability to capture data at multi-scale could give a comprehensive digital data presentation of the materials properties and the actuation performance specification. The machine learning system could be used for the creation of a behavioural digital footprint of a novel material incorporated as a digital library for a rapid process of online material performance referencing and rapid material selection for highly accurate actuation.
